# Pyrvinium pamoate inhibits cell proliferation through ROS-mediated AKT-dependent signaling pathway in colorectal cancer

**DOI:** 10.1007/s12032-021-01472-3

**Published:** 2021-02-08

**Authors:** Wenqian Zheng, Jinhui Hu, Yiming Lv, Bingjun Bai, Lina Shan, Kangke Chen, Sheng Dai, Hongbo Zhu

**Affiliations:** 1 Department of Colorectal Surgery, School of Medicine, Sir Run Run Shaw Hospital, Zhejiang University, 3 Qingchun East Road, Hangzhou, Zhejiang 310016 People’s Republic of China; 2 Key Laboratory of Biotherapy of Zhejiang Province, Sir Run Run Shaw Hospital, Hangzhou, Zhejiang 310016 People’s Republic of China

**Keywords:** Pyrvinium, CRC, ROS, AKT pathway, Cell migration

## Abstract

The use of the anthelmintic drug pyrvinium pamoate (PP) in cancer therapy has been extensively investigated in the last decade. PP has been shown to have an inhibitory effect in colorectal cancer (CRC), but the underlying mechanism remains elusive. We aimed to investigate the antitumor activity and mechanisms of PP in CRC. In the present study, we used CCK-8 assays, colony formation assays, and western blotting to reveal that PP effectively suppressed CRC cell proliferation and the AKT-dependent signaling pathway in a concentration-dependent and time-dependent manner. Flow cytometric analysis and fluorescence microscopy demonstrated that PP increased intracellular reactive oxygen species (ROS) accumulation. We found that the inhibitory effect of PP on cell proliferation and AKT protein expression induced by PP could be partially reversed by *N*-acetyl-l-cysteine (NAC), an ROS scavenger. In addition, the results also demonstrated that PP inhibited cell migration by modulating epithelial-to-mesenchymal transition (EMT)-related proteins, including E-cadherin and vimentin. In conclusion, our data suggested that PP effectively inhibited cell proliferation through the ROS-mediated AKT-dependent signaling pathway in CRC, further providing evidence for the use of PP as an antitumor agent.

## Introduction

Colorectal cancer (CRC) is the third most common cancer among males (after lung and prostate cancers) and the second most common cancer among females (after breast cancer) [1]. Currently, surgery, radiotherapy, and systemic treatment, including chemotherapy, molecular-targeted therapy, and immunotherapy, have significantly improved the survival of CRC patients [2]. Nonetheless, CRC still ranks as the third most fatal cancer globally, and a large proportion of patients have a poor quality of life due to multiple dysfunctions caused by treatments [1, 3]. As an important component of CRC treatment, chemotherapy is characterized by the interference with the cell cycle, injuring not only tumor cells but also normal cells [4, 5]. Therefore, several lines of research on small molecule antitumor agents with reduced toxicity have been investigated in the last few decades [6].

Reactive oxygen species (ROS) are mainly produced in mitochondria during cell metabolism [7]. Different levels of ROS exhibit dual effects on cells [8–10]. At low concentrations, ROS play an effective role in intracellular signal transduction and cellular function regulation [8]. However, excessive increases in ROS levels destroy cellular DNA and gene stability [9]. Cell repair dysfunction and increased oxidative stress contribute to irreversible damage [10]. Compared with their normal counterparts, tumor cells have higher ROS levels [9]. Therefore, cancer treatments targeting ROS may result in fewer side effects for patients [11]. Previous studies have shown that some agents can induce cell cycle arrest and apoptosis by regulating the level of ROS. The elevated ROS levels exceed the antioxidant capacity of tumor cells and lead to cell death [12, 13]. In addition, we also found that ROS-mediated activation of AKT can inhibit cell viability and induce apoptosis [14, 15].

Pyrvinium pamoate (PP) is a classic anthelmintic drug that has been suggested to be a potential agent for cancer therapy in recent years [16]. Esumi et al. showed that PP inhibits cancer cell proliferation and viability during glucose starvation [17]. On the other hand, Wnt signaling promotes the initiation and progression of CRC by causing mutations in adenomatous polyposis coli (APC) and *β*-catenin [18]. As an inhibitor of Wnt signaling, PP significantly suppressed *β*-catenin expression and decreased cell growth by activating casein kinase 1*α* [19]. PP has also been shown to be an inhibitor of the PI3K-dependent signaling pathway, as it effectively suppresses downstream target molecules of PI3K, including AKT and P70S6K [20]. In summary, this agent plays an inhibitory role in various biological processes and signaling pathways in different cancers, delaying tumor proliferation and progression as well as reversing the resistance to therapy [16]. Hence, PP may become a potential adjuvant drug for clinical tumor treatment.

In the present study, we demonstrated the antiproliferative effect of PP and illustrated its potential mechanisms of action on human CRC cell lines. This agent exerts an inhibitory effect through the ROS-mediated AKT signaling pathway. In addition, the small molecule PP could also inhibit the migration of CRC cells.

## Materials and methods

### Cells and cell culture

Human CRC cell lines (HCT116, RKO, and HT29) and normal human colon epithelial cells (Ncm460) were obtained from the American Type Culture Collection (ATCC, Manassas, VA, USA). Cells were maintained in RPMI 1640 plus 10% fetal bovine serum (FBS), 1% glutamine, and 1% antibiotics and cultured at 37 °C in a humidified incubator with 5% CO_2_.

### Chemicals and regents

DMSO used for molecular biology was purchased from Sigma-Aldrich (St. Louis, MO, USA). PP was purchased from Selleckchem (Houston, TX, USA). PP was dissolved in DMSO at a concentration of 2 mM as a stock solution and diluted to the appropriate concentration prior to experiments. *N*-Acetyl-l-cysteine (NAC) was purchased from Sigma (St. Louis, MO, USA). AKT, mTOR, p-mTOR, GSK3β, and p-GSK3β antibodies were purchased from Cell Signaling Technology (Danvers, MA, USA). The Ki67 antibody was purchased from Abcam (Cambridge, MA, USA). E-cadherin, Vimentin, and HRP-conjugated GAPDH monoclonal antibodies were purchased from Proteintech Group (Chicago, IL, USA).

### Measurement of cell viability

Cellular viability was measured with a CCK-8 assay. Cells were seeded at 1 × 10^4^ cells/mL on 96-well plates and incubated for 24 h. Then, DMSO and various concentrations of PP were added to the wells. After 24, 48, and 72 h of treatment, the cells were incubated with 10% (*v*/*v*) CCK-8 solution for 3 h. The absorbance at 450 nm was then measured with a microplate reader. Similar to the method described above, after 72 h of treatment, the cellular viability was determined to calculate the IC50 values. Each experiment was performed at least in triplicate.

### Colony formation assay

Cells were seeded at an initial density of 500 cells/well in six-well plates and cultured with DMSO and pyrvinium. After 2 weeks of culture, the cells were stained with 0.1% crystal violet after fixation in 4% paraformaldehyde. Colonies containing more than 50 cells were measured using a light microscope.

### Transwell migration assay

Cells were suspended and seeded in the upper chamber (~ 1 × 10^4^ cells) and cultured in 100 μL of serum-free medium. The lower chamber was filled with 600 μL of medium containing 10% FBS. After the plates were incubated for 24 h, the lower surface of the plates containing cells was washed, fixed, stained, and imaged. The number of migrated cells was counted in randomly selected fields.

### Wound healing assay

The cells were inoculated in a six-well plate at a density of 1 × 10^6^ cells/well. Then, the cells were allowed to grow to near confluence, and a wound was scratched through the center of the well. After being washed with PBS three times, DMSO and PP were added. Finally, pictures were taken under a microscope after 24 h.

### Measurement of intracellular ROS

We measured ROS levels using an ROS assay kit (Beyotime, Shanghai, China). Cells were incubated with DCFH-DA for 1 h, and the DCF fluorescence intensities were then examined by flow cytometry and fluorescence microscopy.

### Western blot analysis

Cells were lysed in RIPA buffer containing 1 mM phenylmethylsulfonyl fluoride (PMSF) on ice. The lysates were then centrifuged at 12,000×*g* for 10 min at 4 °C. Equal amounts of protein were separated via SDS-PAGE and transferred to polyvinylidene fluoride (PVDF) membranes. Next, the PVDF membranes were blocked with PBST containing 5% skim milk for 1 h and incubated with the indicated primary antibodies, followed by incubation with secondary antibodies. The immunocomplexes were finally analyzed using an ECL system (FDbio).

### Statistical analysis

All graphs and statistical analyses were made using Prism 8 statistical software (GraphPad Software, Inc). The results are given as the means ± standard deviation (SD). Differences were considered statistically significant at values of *P* < 0.05.

## Results

### PP inhibits cell viability

**Fig. 1:**
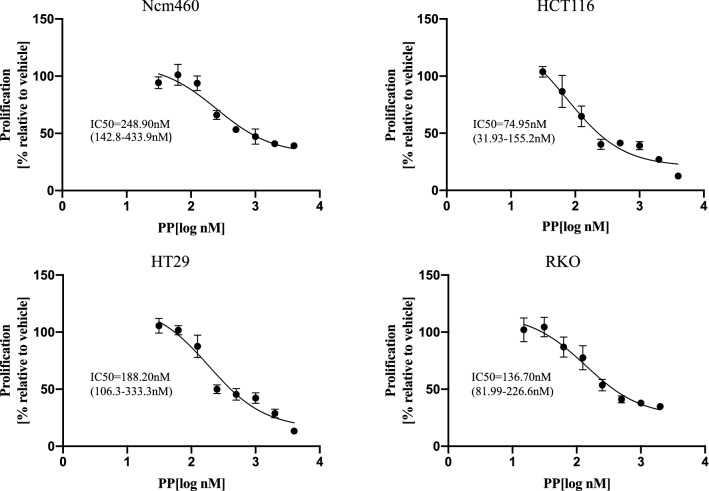
Sensitivity of different colorectal cancer cells and normal human colon epithelial cells to pyrvinium. Three CRC cell lines (HCT116, RKO, and HT29) and one normal human colon epithelial cell line (Ncm460) were treated for 72 h with the indicated concentrations of pyrvinium. The means ± standard deviation are shown

**Fig. 2:**
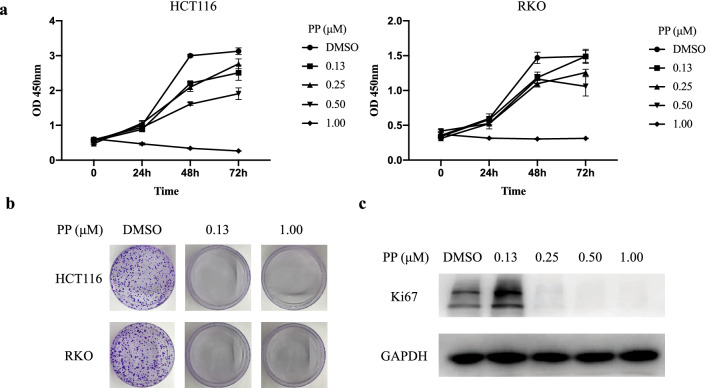
Pyrvinium inhibits cell proliferation in a concentration-dependent manner. **a** The CCK-8 assay was used to measure cell viability after treatment with pyrvinium. **b** Cells were cultured for 14 days with pyrvinium, and stained with 0.1% crystal violet. **c** The protein expression of Ki67 in HCT116 cells was evaluated by Western blotting

The effect of PP on cell viability was examined in three CRC cell lines (HCT116, RKO, and HT29) and one normal human colon epithelial cell line (Ncm460). Cell viability was determined following treatment with different concentrations of PP for 72 h. As shown in Fig. 1, the IC50 values were estimated to be 74.95 nM (HCT116), 188.20 nM (HT29), and 136.70 nM (RKO). The Ncm460 cells demonstrated increased IC50 values of 248.90 nM. Next, HCT116 and RKO cells were selected as the main research objects. The viability of HCT116 and RKO cells was also assessed using a CCK-8 assay after treatment with different concentrations of PP for 24, 48, and 72 h (Fig. 2a). Our data demonstrated that PP decreased cell viability in a concentration- and time-dependent manner. Moreover, the colony formation assay showed that the number of colonies of HCT116 and RKO cells treated with PP was significantly decreased (Fig. 2b). Subsequently, the expression level of Ki-67, which is related to proliferation, was evaluated by Western blot analysis. The results showed that the expression level of Ki-67 obviously decreased after treatment with 0.25 μM PP (Fig. 2c).

### Effect of PP on intracellular ROS

**Fig. 3:**
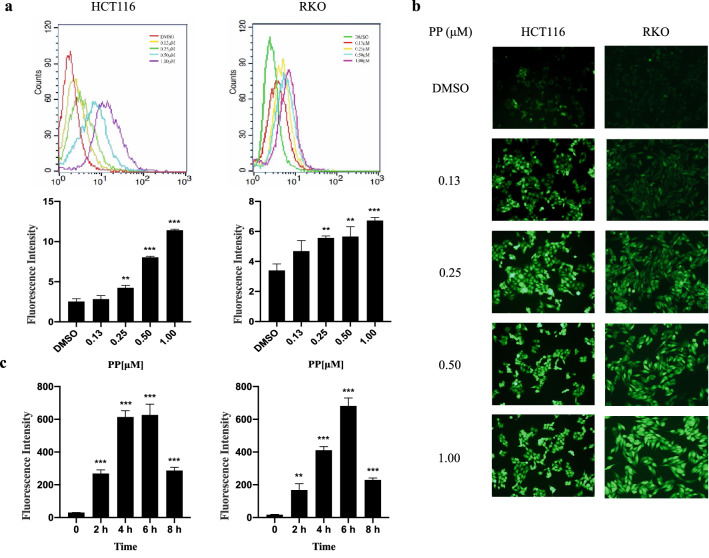
Effect of pyrvinium on ROS in colorectal cancer cells. The level of intracellular ROS was measured by flow cytometry (**a**) and fluorescence microscopy (**b**) after treatment with different concentrations of pyrvinium. **c** After treatment with PP for 2, 4, 6, 8 h, the level of intracellular ROS was measured by flow cytometry **P < 0.01, ***P < 0.001

A variety of studies have established that the excessive accumulation of ROS in CRC plays a vital role in inducing tumor cell death [11]. To examine changes in ROS levels, we measured the oxidative conversion of the sensitive fluorescent probe DCFH-DA to fluorescent DCF. The flow cytometry results showed that the accumulation of intracellular ROS notably increased after treatment with 0.25 μM PP for 24 h. The ROS level of HCT116 cells increased 4.51-fold after treatment with 1.00 μM PP compared with that of the control. Similarly, the ROS level of RKO cells was elevated 1.98-fold (Fig. 3a). Figure 3b shows that the green fluorescence intensity was significantly increased in a concentration-dependent manner in HCT116 and RKO cells treated with different concentrations of PP for 24 h, suggesting an increase in ROS levels. To detect the effect of time on ROS levels, cells were treated with 0.5 μM PP for 2, 4, 6, 8 h. Our results demonstrated that PP has the most obvious effect on HCT116 and RKO cells at 6 h, increasing by 20.73-fold and 40.47-fold, respectively (Fig. 3c).

### Intracellular ROS mediated the inhibitory effect of PP on AKT pathway

**Fig. 4:**
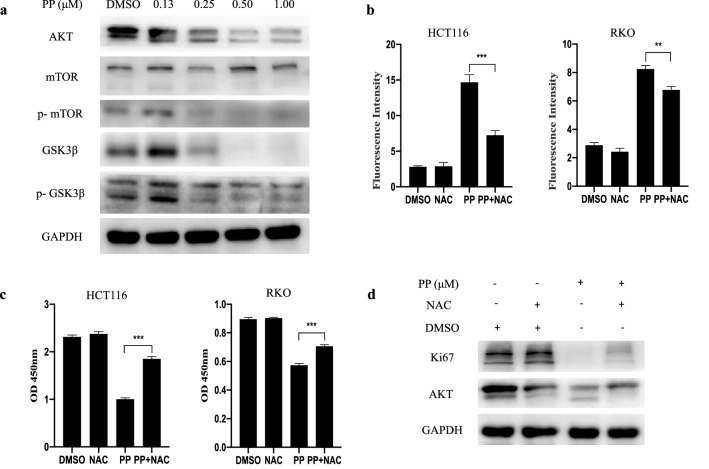
NAC reverses the inhibitory effects of pyrvinium. **a** For Western blot analysis, HCT116 cells were treated with increasing concentrations of pyrvinium for 24 h. Cells were treated with pyrvinium (0.50 μM) in the absence and presence of NAC (5 mM), and then intracellular ROS (**b**), cell viability (**c**), and the expression of AKT and Ki67 in HCT116 cells (**d**) were measured. **P < 0.01, ***P < 0.001

To investigate the underlying molecular mechanism of the antitumor activity of PP, we examined the effect of PP on the AKT signaling pathway. The Western blot results suggested that PP significantly inhibited the expression of AKT, phosphorylated mTOR, GSK3β and phosphorylated GSK3β in a dose-dependent manner but had no effect on the level of total mTOR in HCT116 cells (Fig. 4a). These results indicated that PP inhibited the AKT signaling activity.

Intracellular ROS and cellular viability in response to PP treatment were examined with and without the ROS scavenger NAC (5 mM). In the absence of NAC, treatment with PP-induced ROS accumulation and growth inhibition in HCT116 and RKO cells. As expected, ROS accumulation induced by PP was decreased in the presence of NAC. NAC significantly enhanced PP-suppressed viability (84.31 and 22.90%) in HCT116 and RKO cells, which corresponded to a reduction in ROS accumulation (Fig. 4b, c). Furthermore, we examined protein expression following pretreatment with NAC. The results showed that NAC reversed the inhibitory effect of PP on AKT and Ki-67 expression (Fig. 4d). These results suggest that ROS play an important role in the effect of PP on AKT signaling.

### PP inhibits cell migration

**Fig. 5:**
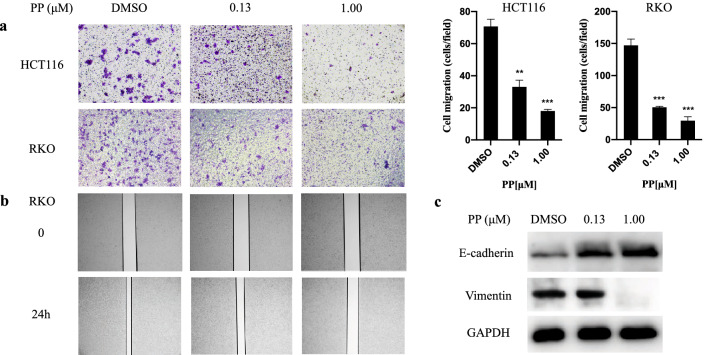
Pyrvinium pamoate inhibits the migration of cells. **a** The migration of cells was observed under an inverted microscope. **P < 0.01, ***P < 0.001. **b** RKO cells were treated with DMSO and different concentrations of pyrvinium. Cells were monitored by microscopy and digital images were captured. **c** The expression of E-cadherin and Vimentin in HCT116 cells was evaluated by Western blotting

Cell migration is intimately linked to tumor metastasis. We used Boyden chamber-type culture inserts equipped with a porous membrane to measure cell migration. Cells were placed into the upper chamber and treated with PP, and migration was evaluated after 24 h. As shown in Fig. 5a, HCT116 and RKO cell migration induced by 1.00 μM PP was reduced by 74.53 and 80.05%, respectively. The wound healing assay was another approach to explore the effect of PP on cell migration. A scratch was applied to the monolayer of RKO cells, and after treatment with PP for 24 h, cell migration was evaluated by measuring the closure of the scratch in the monolayer. Wound healing was significantly wider in PP-treated RKO cells compared to control cells (Fig. 5b). It has conclusively been shown that tumor invasion, migration, and metastasis are associated with epithelial-to-mesenchymal transition (EMT) progression in multiple solid tumors [21]. Therefore, we further analyzed EMT-related proteins, such as E-cadherin and vimentin, by Western blotting. As shown in Fig. 5c, the expression of E-cadherin and vimentin was increased and decreased, respectively, after PP treatment, suggesting that PP could reverse the progression of cancer EMT.

## Discussion

In recent years, the relevance of PP in cancer therapy has been extensively studied [16]. The combination of PP and 5-fluorouracil has a synergistic effect on the inhibition of CRC cell proliferation [22]. PP can also delay tumor growth in pancreatic and breast cancer animal models [17, 23]. Our results demonstrated that CRC cells, especially HCT116 cells, showed lower IC50 values after PP treatment than normal human colon epithelial cells, indicating that PP was an antitumor reagent with low toxicity. In urothelial carcinoma of the bladder, PP could increase DNA double-strand breaks, leading to genomic instability and cell death. The growth of bladder tumors in mice could be suppressed by PP by blocking the cytoplasmic accumulation of HuR [24]. In addition, cancer stem cells (CSCs) have been reported to be the targets of the anticancer effects of PP. PP inhibited mitochondrial oxidative phosphorylation, which is essential for the survival and growth of CSCs [25, 26]. In the present study, we demonstrated that PP inhibited HCT116 and RKO cell proliferation in a dose-dependent manner by CCK-8 assay and colony formation assays. Notably, no colonies formed in the presence of very low PP concentrations (0.13 μM), but the protein expression of Ki-67 did not decrease at this concentration. We suspect that this effect may be related to cell adhesion or intercellular connections and needs to be verified by further studies.

ROS, including superoxide (O_2_⋅−), hydroxyl (⋅OH), peroxyl (RO_2_⋅), and alkoxyl (RO⋅), are associated with numerous physiological and pathophysiological processes in the human body [27]. A study conducted by Liu et al*.* showed that ROS accumulate in the mitochondria of tumor cells and promote cell proliferation, cell survival, cell migration, and epithelial-mesenchymal transition [28]. However, when ROS levels exceed the cellular antioxidant capacity, they contribute to cell damage [13]. A number of studies have explored the antitumor effects of small molecules that modulate ROS in CRC [29, 30]. In our study, the production of ROS increased in a dose-dependent manner after PP treatment and was blocked by NAC. A previous study has shown that increased ROS levels occur in the early stage of apoptosis, followed by the loss of mitochondrial membrane potential [31]. PP treatment caused the ROS levels to increase in a time-dependent manner and reach a maximum at 6 h. Furthermore, our results provided evidence that the accumulation of ROS led to the inhibition of cell proliferation as the inhibitory effect could be reversed by NAC. These findings indicate that the accumulation of ROS occurs at an early stage of inhibition in cell proliferation.

Previous research has shown that the AKT signaling pathway is involved in a variety of biological processes, including growth, proliferation, and apoptosis [32, 33]. Carrella et al*.* showed that PP inhibited breast cancer cell growth by suppressing the activation (phosphorylation) of AKT and P70S6K both in vivo and in vitro [20]. The AKT signaling pathway mediates PP-induced lymphoma T cell (but not normal T cell) apoptosis in hematological malignancies. Additionally, PP has a critical role in suppressing mitochondrial respiration and respiratory complex I activity, leading to elevated ROS and decreased ATP levels [34]. The present study showed that ROS were increased and AKT expression declined in a dose-dependent manner after PP treatment. The findings from these studies suggest that PP can mediate ATK activity and mitochondrial metabolism. Endogenous ROS are mainly produced as a byproduct of mitochondrial respiration [27]. However, the overproduction of ROS in turn leads to mitochondrial DNA double-strand breakage and degradation [35]. A previous study has indicated that arsenic-induced cell transformation through ROS mediates the activation of AKT, ERK1/2, and P70S6K1 [36]. Moreover, ROS have been shown to be related to tumor migration. At a low concentration, capsaicin induces ROS accumulation and promotes CRC metastasis by regulating the AKT/mTOR and STAT-3 pathways [37]. We determined whether PP-induced ROS accumulation was mediated by the AKT signaling pathway, and our results showed that PP inhibited the expression of AKT pathway-related proteins, including AKT, phosphorylated mTOR, GSK3β, and phosphorylated GSK3β. Furthermore, the administration of NAC, the inhibitor ROS, could partially rescue the protein expression of the AKT.

Additionally, our transwell assay and wound healing assay results indicated that PP could effectively suppress CRC cell migration, which would bring a substantial clinical benefit in terms of cancer invasion and metastasis. The EMT plays a major role in increasing cell invasiveness, stem-like features, and resistance to apoptosis, which makes tumors more aggressive [38]. In our study, PP increased and decreased the expression of the EMT-related proteins E-cadherin and vimentin, respectively. Taken together, these results suggest that PP may reverse CRC invasion and metastasis.

There are some limitations in our study. For example, we did not establish an animal model to explore the anticancer mechanism of PP in vivo. Therefore, more in-depth studies and further investigation of the clinical application of PP are needed. However, our present results demonstrated that PP has antiproliferation effects on CRC cells via the accumulation of ROS. Specifically, PP may induce ROS accumulation in CRC cells by decreasing the activity of the AKT pathway. The Western blot results demonstrated that the expression of AKT and the proliferation-associated protein Ki-67 were reversed using the ROS scavenger NAC. These results suggested that PP was related to ROS-mediated AKT signaling. Moreover, PP suppressed cancer cell migration by altering EMT markers. In summary, our findings provide evidence for a deeper understanding of the underlying mechanism of the anticancer effects of PP.

## Data Availability

The datasets used or analyzed in the present study are available from the corresponding author upon reasonable request.
